# The Implications of Regulatory Framework for Topical Semisolid Drug Products: From Critical Quality and Performance Attributes towards Establishing Bioequivalence

**DOI:** 10.3390/pharmaceutics13050710

**Published:** 2021-05-13

**Authors:** Tanja Ilić, Ivana Pantelić, Snežana Savić

**Affiliations:** Department of Pharmaceutical Technology and Cosmetology, Faculty of Pharmacy, University of Belgrade, 11 221 Belgrade, Serbia; tanja.ilic@pharmacy.bg.ac.rs (T.I.); ivana.pantelic@pharmacy.bg.ac.rs (I.P.)

**Keywords:** generic semisolid drug product, extended pharmaceutical equivalence, equivalence with respect to efficacy, qualitative and quantitative composition, microstructure, in vitro release testing, in vitro permeation testing, tape stripping

## Abstract

Due to complex interdependent relationships affecting their microstructure, topical semisolid drug formulations face unique obstacles to the development of generics compared to other drug products. Traditionally, establishing bioequivalence is based on comparative clinical trials, which are expensive and often associated with high degrees of variability and low sensitivity in detecting formulation differences. To address this issue, leading regulatory agencies have aimed to advance guidelines relevant to topical generics, ultimately accepting different non-clinical, in vitro/in vivo surrogate methods for topical bioequivalence assessment. Unfortunately, according to both industry and academia stakeholders, these efforts are far from flawless, and often upsurge the potential for result variability and a number of other failure modes. This paper offers a comprehensive review of the literature focused on amending regulatory positions concerning the demonstration of (i) extended pharmaceutical equivalence and (ii) equivalence with respect to the efficacy of topical semisolids. The proposed corrective measures are disclosed and critically discussed, as they span from mere demands to widen the acceptance range (e.g., from ±10% to ±20%/±25% for rheology and in vitro release parameters highly prone to batch-to-batch variability) or reassess the optimal number of samples required to reach the desired statistical power, but also rely on specific data modeling or novel statistical approaches.

## 1. Introduction

Topical semisolid drug products are among the oldest medicinal dosage forms known to human civilization, widely used in treating a variety of skin diseases. Despite their importance and long history of use, the innovations in semisolid products generally lag behind other pharmaceutical product classes. Since topical products commonly produce lower revenues, the development of both novel and generic products is hindered by the projected return on investment-related risks [1]. Namely, the pharmaceutical industry is to invest significant resources to demonstrate the quality, efficacy, and safety of any product before the authorities grant its market authorization [2]. Semisolid formulations, such as ointments, creams, and gels, due to an extremely complex microstructure (i.e., the microscale arrangement of matter and state of aggregation), are accompanied by more complicated, interdependent relationships among the structure, properties, manufacturing process, and performance as compared to solid and injectable dosage forms, that increase the potential for variability and number of failure modes [3,4]. Furthermore, topical drug products face unique obstacles to the development of generics compared to other drug products for which the assessment of bioequivalence is amenable to traditional pharmacokinetic methods [1,5].

As the target site of the most topical semisolid formulations is either the skin or the underlying tissue, due to the none or very low measurable amounts of drug in the systemic circulation, traditionally, establishing bioequivalence in most cases has been based on comparative clinical trials, which are time consuming and expensive, but more importantly, often associated with a high degree of variability and low sensitivity in detecting formulation differences [5,6,7,8,9]. In general, clinical trials require the demonstration of bioequivalence of the prospective generic to the reference/comparator drug product, using one or more clinical endpoints and guaranteeing efficacy by establishing superiority of the tested formulation over a placebo [10]. A clinical response to topical drugs is quite variable due to the numerous patho-physiological factors as well as difficulties involved in the standardization of the applied dose [9]. Likewise, in such cases there is no true placebo since the vehicle components also exert some effects, making the primary endpoints of clinical trials more difficult to meet [2]. As a result, despite the enrollment of a large number of patients in clinical trials (*n* > 500), frequently, the formulation differences cannot be efficiently detected [10]. This represents a substantial challenge to generic manufacturers and an additional cost for the patients [9]. Indeed, in U.S., in the 2011–2015 period, a significant price increase (almost 276%) was observed for topical generic products, while, simultaneously, oral generic drugs demonstrated a price decrease (21%) [1]. In order to improve the patient access to more affordable topical semisolid drug products on the market, demonstration of bioequivalence requires the usage of alternate approaches which are faster, less expensive, more reproducible, and sensitive to differences in topical products [1,9].

In this context, firstly, to optimize the regulatory requirements for the therapeutic equivalence of topical semisolid drug products, pharmaceutical scientists and dermatologists from academia, industry and regulatory agencies, have proposed the Strawman decision tree and the topical drug classification system [9,10]. Both these approaches highlighted the importance of accounting product specificities, including the properties of pharmaceutical form, drug, site of action and indication. The information on qualitative/quantitative composition and microstructure of the semisolid products being compared represents the basis for rational selection of relevant in vitro/in vivo product performance measures for the determination of bioequivalence [5,9,10,11]. As a result, within the last few years, both European and American regulatory authorities have been advancing regulation relevant to topical generic products, accepting different non-clinical, in vitro/in vivo surrogate methods for topical bioequivalence assessment [12]. From 2012, U.S. Food and Drug Administration (FDA) has continuously published non-binding, product-specific guidelines for generic product development, to identify the appropriate methodology for developing drugs and generating evidence needed to support abbreviated new drug application (ANDA) approval [13]. Over the past five years, a number of relevant guidelines were made public, including an in vitro option to establish bioequivalence of topical semisolid drug products [10,11] (Table 1). As outlined in Table 1, specific in vitro tests that should be performed to support a claim of therapeutic equivalence, in lieu of clinical endpoint studies, highly depend on intrinsic complexity of a specific product.

**Figure 1:**
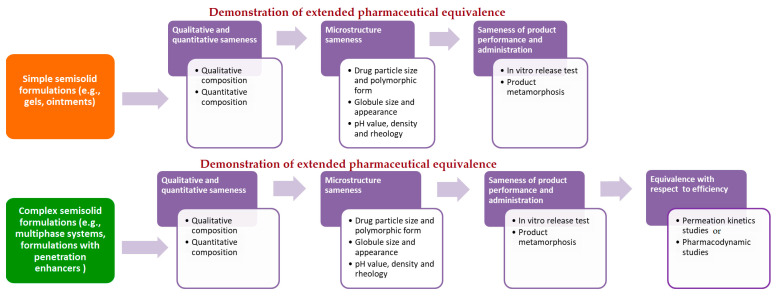
Schematic illustration of the proposed regulatory framework for bioequivalence assessment of topical semisolid drug products according to recently issued EMA draft guideline.

On the other hand, in October 2018, European Medicines Agency (EMA) published for public consultation a universal guideline for topical generic product submission entitled *Draft Guideline on Quality and Equivalence of Topical Products*. Due to the high diversity of topical products, the complex range of skin conditions that should be treated and the variety of patient needs, this guideline does not provide a single procedure, but states that general recommendations should be adopted on a case-by-case basis [14]. Despite the obvious differences in the manner of proposing the recommendations for generic drug development, EMA requirements are generally similar to those of the FDA. Precisely, according to EMA draft guideline, in case of simple semisolid formulations (e.g., gels, ointments), therapeutic equivalence can be extrapolated from the comparative quality data with the relevant comparator medicinal product (extended pharmaceutical equivalence concept). For this purpose, comparative analysis of pharmaceutical form, qualitative and quantitative composition, microstructure/physical properties, product performance and administration should be performed. In case of complex formulations (e.g., multiphase systems) or those comprising excipients that might affect drug bioavailability and performance, an additional biorelevant test, such as permeation kinetics (in vitro skin permeation, tape stripping or pharmacokinetic bioequivalence) or pharmacodynamic (vasoconstriction assay for corticosteroids or tests relevant for antiseptics and anti-infectives) studies, should be employed (equivalence with respect to efficacy concept) [14] (Figure 1).

However, it should be noted that the proposed EMA draft guideline is the subject of intensive academia and industry-wide discussions, seeking reliable and robust surrogate bioequivalence methodologies. Despite the significant advances made in the development of generic semisolid products, several limitations have been identified, restricting its successful translation into practice [15]. Therefore, this review intends to provide a comprehensive insight into the implications of the proposed regulatory framework for equivalence demonstration for generic semisolid products, analyzing the more recent data obtained during extended pharmaceutical equivalence characterization, as well as efficacy equivalence studies. The strengths and limitations of each regulatory accepted method are presented in brief, including their suitability for abridged bioequivalence demonstration of generic semisolid drug products. Particular attention was given to solutions proposed for constraints hampering the applicability of currently available regulatory guidelines in practice.

## 2. Demonstration of Extended Pharmaceutical Equivalence of Topical Semisolid Drug Products

### 2.1. Evaluation of Qualitative (Q1) and Quantitative (Q2) Sameness

Drug delivery at the target skin site from topical semisolid products is a complex phenomenon, which depends on the drug physiochemical properties, the disease state and in particular, formulation effects [9]. The formulation composition (excipients’ nature and concentration) is crucial for the therapeutic efficacy, since it directly affects drug solubility and thermodynamic activity, drug release from the dosage form, skin barrier properties and drug penetration/diffusion into/through the skin [16]. Therefore, both European and American regulatory authorities require the demonstration of acceptable Q1 and Q2 sameness, i.e., to document that the test product contains the same excipients in the same quantitative composition as the comparator medicinal product (differences not greater than ±5% are acceptable). According to EMA draft guideline, only excipients whose function is not related to product performance and administration (i.e., antioxidants, preservatives, coloring agents) could be qualitatively and quantitatively different (not more than ±10% is acceptable) [14]. Since the excipients in the comparator product are listed in the patient information leaflet, establishing the Q1 sameness seems to be relatively simple. On the other hand, in order to achieve Q2 sameness, reverse engineering of the comparator product needs to be performed, applying appropriate and validated analytical methods [2,15]. However, due to patent pending or undesirable quality outcome, manufacturers of generic semisolid products are sometimes compelled to modify the formulation composition of the comparator product, and consequently, accomplishing the Q1/Q2 sameness could be a quite challenging task [15,17]. Additionally, as stated in EMA draft guideline, not only formulation composition, but also, the grade of the excipients should be the same, due to its significant impact on the product quality and performance [15,18,19]. For example, analyzing the effect of 6 different petrolatum sources on drug product performance containing petrolatum as the only vehicle, it was observed that diverse grades of petrolatum produced significantly different release rates of a topical steroid, due to variations in the distribution ratios of the hydrocarbons chain lengths [19,20]. However, the grade of excipients used in a comparator product is only available to the regulatory agencies. It is quite demanding to experimentally analyze the grade of any excipient within semisolid formulations, and therefore, assuring the sameness of excipient grades could be difficult to achieve for most generic manufacturers [15].

Although demonstration of Q1/Q2 sameness is considered critical in reducing the failure modes related to the product performance, the variations of key functional excipients, even within the acceptable range (±5%, *w*/*w*), can significantly affect the drug bioavailability. In this regard, the results of a recent study performed by Kumar Sharma et al. [21] deserve to be mentioned here, since it investigated the effects of incremental changes in the surfactant concentration (±5%, *w*/*w*) on the quality and performance attributes of metronidazole-loaded cream products that meet the definition of Q1/Q2 sameness. Although the monitored quality attributes (globule size, rheology, pH, water activity, rate of drying) practically overlapped, in vitro permeation profiles were remarkably different between the tested formulations. Acceptable 5% *w*/*w* change in surfactant concentration obviously led to significant change in the degree of drug saturation during product evaporative metamorphosis, ultimately influencing its performance [21]. This study confirmed that the change in drug thermodynamic activity during metamorphosis, due to slight variations in formulation composition, could significantly alter the drug bioavailability. Although EMA draft guideline asserts that for volatile solvent based topical products, product transformation on administration should be also compared, no methodologies have been proposed for this purpose [14,15]. Therefore, again, the requirement regarding product metamorphosis sameness proves to be difficult to attain. In other words, although different methods have been proposed in the literature (e.g., ATR-FTIR spectroscopy, localized nanothermal analysis and photothermal microspectroscopy combined with multivariate data analysis) [15], there are still limited data on their applicability for the characterization of a wide range of topical semisolid products. Therefore, it is essential that EMA provides more detailed recommendations for studying the product metamorphosis.

### 2.2. Comparative Characterization of Critical Quality Attributes (CQAs)

Although the criteria for Q1/Q2 sameness are met, due to complex formulation composition and manufacturing process parameters, a generic semisolid product may exhibit differences in the microstructure and arrangement of matter compared to the reference product, that may impact its quality and performance attributes [6,17,22]. Various factors determine specific product microstructure, such as size and shape of dispersed particles, polymorphism, agglomeration, droplet size of the internal phase, excipients’ source/grade, processing conditions and storage [17,22,23]. Therefore, according to the EMA draft guideline, for the demonstration of extended pharmaceutical equivalence, comparative characterization of microstructure/physical properties should be performed, analyzing the CQAs that can influence drug bioavailability, usability or can indicate inconsistency in the manufacturing process and product stability issues. For semisolid formulations, pH value, density, and rheological behavior are identified as the main risk factors that should be closely monitored to gain an assurance of microstructural similarity. For suspension and immiscible phase formulations, additional characterization in terms of drug particle size distribution and polymorphic form, that is, globule size distribution and appearance is required [14]. The similar requirements are set out in the FDA product-specific guidelines containing an in vitro option of bioequivalence assessment. Physicochemical characterization should include comparative analysis of appearance, rheological properties, drug particle size and size distribution, globule size, pH, water activity, and other potentially relevant physical and structure similarity characteristics [11,13]. However, it should be noted that the reliable characterization of microstructure has sparked numerous discussions among different stakeholders (academia, industry and several regulatory agencies) during the last few years. Among other, they imposed the following two questions: (i) which quality attributes are truly critical to the therapeutic performance of topical semisolid dosage forms, as well as (ii) what are the appropriate methodologies for measuring each of these quality attributes without disturbing the original product microstructure [3,4]. Currently, both European and American regulatory authorities do not provide recommendations for the methods that should be utilized for measuring the mentioned CQAs.

Generally, the rheology of semisolid products is highly sensitive to alternations in the product microstructure, and therefore, detailed rheological characterization takes the central role in detection of the potential microstructure differences [22,24]. Furthermore, rheological characterization serves as a useful quality and stability indicator, which could provide additional information concerning batch variability, product sensorial properties (e.g., consistency, spreadability, and feel) and consequently patient compliance [22,25]. Hence, EMA defines specific rheological parameters that should be documented when characterizing the rheological profile of a given formulation. Precisely, (i) a complete flow curve of shear stress (or viscosity) versus shear rate, (ii) yield stress, and (iii) the linear viscoelastic response (storage and loss modulus vs. frequency) should be determined. Additionally, the product’s behavior should be classified according to shear and time effects and described using appropriate metrices (viscosities at specified shear rates across the rheograms (e.g., η100); plastic flow yield stress values; thixotropic relative area (SR); viscoelastic storage and loss moduli (G’ and G”); apparent viscosity; loss tangent (tan δ)) [14]. These parameters should be determined in at least three batches of the test and reference products with at least 12 replicates per batch. In order to declare microstructure equivalence, the 90% confidence interval (CI) for the difference of means of the test and reference products should be included within the acceptance limits of ±10% of the reference product mean, assuming normal distribution of data [14]. This requirement has been intensively disputed in the literature during the last two years as overly restrictive, because it does not take into account the intrinsic variability of topical semisolids [15]. In an attempt to clarify this issue, Pleguezuelos-Villa et al. [23] compared rheological data of Q1/Q2 equivalent test and reference diclofenac diethylamine-loaded emulgels with the results obtained from in vivo pharmacokinetic study in 32 healthy volunteers. Despite statistically significant difference in rheological parameters (90% CI was outside the 90–111% limits), the investigated products could be considered bioequivalent based on the in vivo bioavailability assay. This finding suggests that a difference beyond ±10% between rheological parameters of test and reference products does not necessarily translate into relevant in vivo differences [23]. Similarly, while analyzing the spreadability of three generic formulations that were shown to be equivalent to the innovator product during clinical bioequivalence studies, Kryscio et al. [24] observed that the equivalence in spreadability (inversely proportional to yield stress) is not a prerequisite for product bioequivalence.

In this regard, it should be emphasized that before EMA draft guideline became available for public consultation, all rheological parameters listed above were not a part of routine analysis when releasing new bathes, and therefore, limited data regarding the batch-to-batch variability was available [26]. Hence, Mangas-Sanjuán and coworkers [26] performed comprehensive rheological characterization of 10 batches of a reference product (Daivobet^®^ ointment 50 µg/0.5 mg/g, Leo Pharma A/S, Ballerup, Denmark, containing calcipotriol and betamethasone) to evaluate whether the inter-batch variability of the rheological parameters allows demonstrating equivalence within a ±10% acceptance range. Analyzing the obtained 90% CIs (based on both parametric and non-parametric data analysis), the equivalence for most of the rheological parameters could not be demonstrated. In other words, due to the relatively high inter-batch variability (>10% for several parameters), an acceptance range of ±10% was inappropriate to declare quality equivalence [26]. Generally, the observed high batch-to-batch variability can be derived from the complexity of excipient source (excipient intra-supplier variability), small differences in manufacturing procedure, batch size, storage conditions and aging of the formulations [26,27]. Therefore, in order to overcome the observed limitations of rheological measurements, the authors proposed (i) to widen the acceptance range up to ±20% (which corresponds to those for AUC and Cmax in pharmacokinetic bioequivalence studies) or (ii) to calculate the optimal number of batches required to reach the desired statistical power based on the batch-to-batch variability [26]. Similarly, while characterizing three batches of eight reference blockbuster semisolid drug products in the EU market, Miranda et al. [27] observed that none of the same product batches could be considered as equivalent according to EMA criteria, due to the high variability in rheological parameters (at least two rheological endpoints were statistically different between the batches of the same product). This clearly confirms the need for establishing new microstructure sameness criteria, taking into account the intrinsic variability of the product being studied [15,27]. In this context, Xu and coworkers [28] tried to establish the optimal number of batches and replicates per batch based on different scenarios of inter-batch and intra-batch variability, to accurately demonstrate microstructure similarity between two semisolid products. The calculation of proper sample size is important to disable data manipulation by preventing pharmaceutical companies to choose those product batches that behave similarly. Founded on the simulation-based data analysis, it was concluded that, in cases of low intra- and inter-batch variability, the minimum number of batches should be three, with minimum six units per batch. For the products with up to 5% difference, testing six batches with 12 units per batch or three batches with 24 units per batch could be sufficient to declare equivalence. Finally, in cases when intra- or inter-batch variability exceeds 10%, the number of batches and/or the number of units should be further increased [28].

Additionally, it should be emphasized that usual approach for calculation of CI for the difference of means of the test and reference product, relative to the reference product mean, does not consider the variability in the reference mean estimate [29]. Hence, assuming normal data distribution, Ocaña and collaborators [29] proposed new CI for the test/reference mean ratio, based on the Fieller’s theorem, which takes into account both the within-batch and the between-batch variance, thus enabling more accurate equivalence declaration. Due to the relatively large number of rheological parameters that should be tested as well as high restrictiveness of EMA draft guideline, it was not possible to demonstrate equivalence even between two packaging formats of the same reference product (betamethasone ointment 0.5 mg/g). Hence, for multivariate concepts, such as rheology, Ocaña et al. [29] also suggested to summarize all of the continuous variables to just one or a few variables by means of principal components analysis technique (PCA) (for more details, please see Ocaña et al. [29]). Additionally, several studies noticed that rheological parameters frequently do not follow normal distribution. Therefore, the calculation of 90% CI based on the ratio of geometric means of test and reference products seems to be more appropriate [23,26,29].

On the other hand, from a regulatory point of view, the prerequisite for use of rheology methods as a tool for microstructure characterization of topical semisolids either for quality control or equivalence demonstration is an appropriate standardization of the procedure. However, currently, there are no regulatory recommendations for the standardization, i.e., formal validation of this method. Hence, Simões and coworkers [25] tried to establish a practical approach for validation of the rheological analysis, including the rheometer qualification and the validation of numerous operational critical parameters for a rheology profile acquisition. The experimental results showed that the rheology measurement method can be successfully validated, proving its suitability to determine sameness/differences between the formulations. Likewise, obtained findings inter alia showed that geometry configuration, sample application mode and temperature are critical method variables that should be carefully optimized before each analysis. According to the risk assessment analysis, the thixotropic relative area, oscillatory yield point, flow point, and viscosity related endpoints were defined as highly sensitive and discriminatory monitoring responses [25]. Hence, it is believed that the early inclusion of rheological measurements in product manufacture would allow identifying the factors responsible for microstructure variations, which in turn would assure the satisfying product quality and reduce the overall batch variability [25].

For immiscible phase formulations, such as creams, globule size may directly affect the product stability and performance. Poor control of globule size may result in phase separation, creaming or cracking of the semisolid products [30]. On the other hand, the alterations in globule size among the prospective generic and reference semisolid drug products may impact the amount of drug entrapped in the globule, its partitioning between the oil and water phase, and consequently, drug release and partitioning into the skin [30]. For the given combination of excipients, manufacturing process parameters (e.g., rate of mixing, temperature, order of excipients addition) may significantly impact the globule size [30,31]. All these considerations imply the need for careful monitoring of globule size to ensure the microstructure sameness. However, recent studies imposed several conclusions: (1) globule size can significantly vary from the batch to batch of the same semisolid drug product, (2) differences in globule size do not always correlate with differences in rheology or release profile, and (3) even if EMA criterion for globule size sameness is not fulfilled, two products can still be bioequivalent (as confirmed in human in vivo bioequivalence study) [23,27]. Moreover, it is important to highlight how challenging it may be to analyze the globule size of semisolid products. The characterization of emulsion droplets is usually performed using optical microscopes coupled with appropriate software analysis of the globule size distribution (e.g., using free image-analysis software like Image J, National Institutes of Health, Bethesda, MD, USA), although other techniques have also been proposed (e.g., morphologically directed Raman spectroscopy, laser diffraction) [32]. Generally, the microscopic analysis requires the measurement of thousands of particles to obtain statistically valid particle size distribution [32]. Simultaneously, this analysis is associated with high variability (e.g., coefficient of variation (CV) of almost 38.91% according to Pleguezuelos-Villa et al. [23]) and requires careful standardization of the procedure for sample preparation.

Many failure modes of generic semisolid drug products arise from the differences in the physical and structural properties of the drug compared to the reference product. Generally, the variations in drug particle size, morphology and polymorphic form may affect both bulk qualities (such as rheology, density, content uniformity, and other physical properties) and product performance (such as drug release and efficacy of drug delivery to the target site) [3]. Indeed, recently, it was observed that the size of drug particles was one of the main factors determining acyclovir release from cream formulations [33]. As authors emphasized, particle size of the dispersed acyclovir is the CQA that should be carefully controlled when developing acyclovir topical creams with desired performance characteristics [33]. However, it is quite difficult to ensure the same drug particle size and morphology in the prospective generic product as in the reference product, because they are highly dependent on the properties of the raw drug. Although milling of the raw drug can help reduce the particle size and thus obtain comparable sizes to the reference, the ultimate particle size depends on the solubilization effect of the cosolvents/surfactants used in the formulations and/or the shearing effects during the homogenization process of the creams. On the other hand, unlike drug particle size and morphology that can be relatively easy determined using the microscopic techniques, the characterization of drug-specific polymorph requires more sophisticated techniques like X-ray diffractometry, thermal analysis, or others. It can be technically quite difficult to analyze the polymorphic form in semisolid products, due to the risk of form conversion, including crystallinity change, during the sample preparation [32].

A formulation’s pH value may have considerable influence on drug solubility, ionization state, polymorphic state, ratio of dissolved to undissolved drug, amount of drug in the phase in contact with the skin, as well as a formulation’s viscosity and stability, thus determining the product quality and performance [4]. Likewise, safety and local tolerance of topical semisolid products may be affected by their pH value, since application of a topical formulation with pH that markedly deviates from the skin pH may cause irritation, particularly when accompanied with a skin condition/disease [30]. Considering that the final product’s pH value is governed by the inherent nature of the drug, excipients interactions within the formulation, and also by the manufacturing process (e.g., order of components’ addition) [30,31], it is clear that pH, as a CQA, should also be monitored for the demonstration of extended pharmaceutical equivalence. For example, in acyclovir cream products, the soluble fraction of acyclovir in the aqueous phase has been identified as the critical factor for the product performance and its therapeutic outcome. Since acyclovir has two pKa values (2.27 and 9.25), depending on the pH of the aqueous phase, soluble fraction of acyclovir may be present in cationic, zwitterionic, and anionic forms, which may have different skin permeation potential [7]. In this context, recently, Kamal and coworkers [33] investigated the effects of different formulation variables (propylene glycol, poloxamer and sodium lauryl sulfate concentrations) and different pHs of the aqueous phase (4, 6.5, 9) on critical quality and performance attributes of acyclovir cream. Interestingly, the intentional change in pH of the aqueous phase did not significantly affect acyclovir final concentration in the aqueous phase, and consequently had negligible effect on acyclovir permeation and skin retention. It appears that other excipients involved (predominantly propylene glycol) masked the effect of pH on ionization of acyclovir molecules and their delivery into and through the skin in vitro [33]. Additionally, it should be noted that, while analyzing pH values of three batches of eight reference semisolid drug products, Miranda et al. [27] observed significant inter-batch differences in the pH value, despite the same formulation and processing conditions. Although, undoubtedly, the same composition and microstructure attributes (inter alia pH values) related to the comparator product can help ensure the same therapeutic performance of the prospective generic, both mentioned studies again impose the conclusion that acceptance limit (90% CI within ±10% of the reference product mean) proposed by EMA for pH sameness is too restrictive, i.e., more reasonable criteria should be specified.

Finally, according to EMA draft guideline, comparative analysis of density, as another important quality attribute, should also be performed during microstructure characterization for abridged bioequivalence demonstration. Density of a sample directly affects the dose withdrawn and applied by patients—the lower dose will be drawn from the formulation with lower density compared to high density one [34]. However, unlike rheological properties that have been the subject of various studies during the last few years, literature data whether and how the variations in density of Q1/Q2 equivalent topical semisolid products affect the product performance are still lacking. Consequently, since acceptance criteria for a generic product, according to EMA draft guideline, are ultimately dependent on reference product results [27], detailed investigation of batch-to-batch variability of density is needed.

### 2.3. Evaluation of Product Performances—In Vitro Release Test

The release of drug from topical semisolid dosage forms directly affects the onset, duration and magnitude of therapeutic response, since drug has to be liberated before being available to the skin. On the other hand, drug release kinetics highly depend on the combined effect of several physical and chemical parameters of semisolid products, such as solubility and particle size of the drug, method of drug distribution within the formulation and rheological properties [10,17,35]. Although not a direct indication of drug bioavailability, an in vitro release test (IVRT), using diffusion cells and synthetic membranes, can discriminate the differences in drug release rates arising from the formulation changes and various physicochemical properties of the semisolid drug products and consequently, can signal inadequate in vivo performances [35]. Hence, IVRT has been recognized as a valuable tool at various stages of the generic topical product development (early formulation development phase, scale-up, batch-to-batch consistency, life cycle management, post authorization changes) [36,37]. As a result, EMA draft guideline define the release rate as a CQA to be specified in the finished product release and shelf-life specification (unless otherwise justified). Additionally, a validated IVRT, as a method for product performance characterization, is required to support extended pharmaceutical equivalence [14]. Here, it should be noted that 1997 FDA Scale Up and Post Approval Changes for Nonsterile Semisolid Dosage Forms guidance also recommended IVRT to assure consistent product performance during the post-approval period after an acceptable level of changes in (i) the component or formulation composition, (ii) the manufacturing process and equipment, (iii) scale-up/scale-down of manufacture, or (iv) the site of manufacture of semisolid products [38]. More recently, in appropriate product-specific guidelines for generic drug development, the FDA continues to recommend the use of IVRT to support the evaluation of bioequivalence [13,35,36].

In order to discern potential differences between the test and comparator products, the following experimental conditions should be carefully selected: (a) membrane type, (b) composition of receptor medium, (c) test duration, sampling time and experimental conditions (such as apparatus, temperature, mixing speed), (d) the amount and method of formulation application and (e) analytical method for quantifying the amount of drug in the receptor solution [14]. Ideally, a synthetic membrane should act as an inert support that separates the drug product from the receptor medium without binding the drug, while simultaneously providing minimal resistance to its release [14,39]. Generally, there are numerous reports in the literature describing the influence of membrane material on the drug release (e.g., [39,40,41,42]). Considering the obtained findings, it appears that membrane selection is at least in part drug/formulation dependent, and there are still no useful recommendations for the selection of the most suitable membrane type for IVRT. Here, it is interesting to note that Mekjaruskul et al. [39], while analyzing the influence of membrane type on dexamethasone release, observed that the same membranes with similar average pore size may yield different release profiles if acquired from different suppliers. This finding suggests that inter-supplier variability should be also taken into account during the membrane selection. 

Similarly, the receptor medium should be carefully selected in order to maintain sink conditions during the release experiments [14]. For this purpose, the investigation of drug solubility in different receptor media should first be performed. Considering that this step can be quite time consuming, recently, in silico studies using Chemaxon^®^ software (ChemAxon, Budapest, Hungary) were suggested to rationalize the selection of solvents suitable for solubility studies, based on respective chemical descriptors (such as size, geometry, lipophilicity, solubility, and surface topology) arising from drug chemical structure [37]. According to the EMA draft guideline, the duration of IVRT should be sufficient to properly characterize the release profile. Ideally, at least 70% of the drug applied in the donor chamber should be released [14]. However, in vitro release data obtained during the characterization of eight different topical semisolid drug products indicate that no more than 50% of the drug tends to be released during the 24-h study [27]. Similar trend was also observed in several other studies which utilized IVRT to assess the performance of topical semisolid drug products (e.g., [7,43]). Considering that prolonged assay duration does not mimic in vivo conditions, it is imposed that EMA should reconsider this requirement [15]. In addition, according to the EMA draft guideline, 12 replicates with at least six sampling time points within the linear portion of the release profile are required to thoroughly characterize release process for each product [14]. Subsequently, hundreds of samples are generated throughout IVRT studies, requiring rapid analysis of drug content to avoid stability issues. In this sense, aiming to assist manufacturers of generic topical semisolid products, Miranda et al. [37] recently established a portfolio of reversed-phase high-performance liquid chromatography (RP-HPLC) methods specifically tailored for commercially available topical products for a real-time drug analysis of the samples generated during the IVRT.

According to recent regulatory requirements, during the marketing authorization procedure, adequate evidence should be provided to document that IVRT method is properly validated. The requirements of European and American regulatory authorities are similar, but significantly more details regarding procedure validation can be found in appropriate FDA product-specific guideline (*Draft Guidance on Acyclovir*) [44]. The basic concepts for validation of the IVRT method are presented in Table 2. Since 2018, an increasing number of studies deals with validation of IVRT method for different semisolid drug products (e.g., acyclovir cream [35], diclofenac emulgel [36], miconazole nitrate cream [45], hydrocortisone acetate cream [46], metronidazole cream [47]). The first comprehensive report in scientific literature on the successful qualification/validation of IVRT was published by Tiffner and coworkers in 2018 [35]. This study is particularly valuable because it provided detailed procedures for (i) qualification of the IVRT apparatus operational parameters (receptor chamber capacity, orifice diameter, temperature control, stirring speed, dispensed sampling volume and environmental conditions), (ii) qualification of the laboratory’s capability to perform IVRT studies and (iii) validation of HPLC method for drug quantification, which are stated, but not explicitly described in FDA product-specific guideline [44]. However, as authors emphasized in the manuscript, the data regarding IVRT method development (which are required according to both European and American regulatory agencies) are lacking [35]. Generally, the omission of IVRT method development and validations reports is considered to be among the main factors impairing the approval of generic semisolid drug products [36]. Hence, to reduce the time required for method development along with the overall costs, Miranda et al. [36] proposed an analytical quality by design (aQbD) approach for development of IVRT method. In brief, after establishment of an analytical target profile, through the risk assessment analysis, the critical analytical attributes (in vitro release rate, cumulative amount released at initial/final time point and dose depletion) and critical method variables (receptor medium, membrane and dose regimen) were identified. Based on results of a 3 × 2 × 3 factorial design, the most suitable IVRT parameters were chosen and the comprehensive validations studies of IVRT was further performed, following the EMA and FDA requirements [36].

Finally, for the demonstration of extended pharmaceutical equivalence, EMA set the following requirement: “the 90% confidence interval for the ratio of means of the test and comparator products for the parameters (R), (A) should be contained within the acceptance interval of 90–111%” [14]. However, it is important to emphasize that FDA sameness criterion is not too strict, i.e., 90% CIs of in vitro release rate should be within 75–133.33% [38,44]. As a result, taking into account these requirements, an increasing number of publications during the last few years has been investigating the sensitivity and discriminatory capability of IVRT for demonstration of sameness or difference between semisolid drug products. Recently, while analyzing in vitro performances of Q1/Q2 different test (Calcipotriol/Betamethasone Sandoz^®^, Lek Pharmaceuticals d.d, Ljubljana, Slovenia) and reference (Daivobet^®^, LeoPharma A/S, Ballerup, Denmark) ointments containing fixed combination of calcipotriol and betamethasone, Habjanič et al. [48] observed significantly higher release rates of both drugs from the test compared to the reference product. However, the results of clinical study (conducted on 444 male and female adult patients) showed that differences between these products were not clinically significant and both products were concluded to be therapeutically equivalent for the topical treatment of plaque psoriasis vulgaris. Similarly, while comparing the in vitro drug release rates from three batches, for each of the eight selected reference semisolid products, Miranda et al. [27] noticed that none of the batches of corresponding product exhibited 90% CI within the EMA acceptance limits (90–111%), due to high inter-batch variability. Contrary to that, when wider FDA criteria were applied, the majority of product batches could be considered as equivalent. These findings clearly underline that EMA should specify more reasonable criteria for product sameness, considering the intrinsic variability of topical semisolid dosage forms [27].

## 3. Demonstration of Equivalence with Respect to Efficacy of Topical Semisolid Drug Products

The complexity of the release mechanisms, the active role of several excipients in the skin penetration of a given drug, and the changes induced by their interaction with the biological barrier prospectively restrict the bio-relevance of an IVRT, performed under infinite dose conditions using synthetic membranes [17]. Therefore, as emphasized in the introductory section, according to EMA draft guideline, in the case of complex formulations (such as multiphase systems) or those containing the excipients whose function is to influence drug bioavailability and product performance (e.g., chemical penetration enhancers), additional permeation kinetic or pharmacodynamic equivalence data is required for the demonstration of bioequivalence [14]. Unlike in vivo pharmacodynamic vasoconstrictor assay that has a long history of use, being recommended by the FDA (*Guidance Topical Dermatological Glucocorticoids: In vivo Bioequivalence* (1995)) [49] and other major regulatory authorities for topical corticosteroid drug products, the acceptance of dermal pharmacokinetic-based approaches (in vitro permeation testing and tape stripping) by EMA represents significant progress in regulatory science. Although according to the Strawman decision tree, confocal Raman spectroscopy and microdialysis have been proposed as alternative methodologies to study drugs with the target site of action in the stratum corneum (SC) and epidermis/dermis, respectively [9], EMA emphasized that these techniques are not sufficiently established to provide pivotal equivalence data, but may be utilized as a support [14].

### 3.1. In Vitro Permeation Test

The utility of in vitro permeation test (IVPT) methodology for the documentation of bioequivalence has been supported by substantial body of evidence showing that in vitro results correlate well with and are predictive of human in vivo bioavailability data [5,50]. Likewise, numerous studies confirmed the capability of IVPT methodology, if properly conducted, to provide the same conclusions as in vivo clinical endpoint studies regarding the bioequivalence of two semisolid drug products [5] (for details, please see Raney et al. [5]). The human skin retains its barrier properties for percutaneous absorption of different drugs following the excision from the body, and therefore, is recommended as a membrane to establish product equivalence with respect to efficacy [15]. However, it is important to emphasize that although this methodology has been used for almost half of a century in in vitro drug penetration/permeation studies, it was not accepted by EMA for the evaluation of topical semisolid products until 2018, due to difficulties in the procedure validation. Namely, due to high variability of human skin (related to gender, race, age and anatomical site), the method standardization and verification of reproducibility is a quite challenging task [51]. Therefore, to manage the variability, EMA provides certain, generalized recommendations for: (i) membrane choice (inclusion/exclusion skin sections, skin preparation techniques, skin integrity, number of skin donors and replicates per donor), (ii) choice of receptor medium (composition, criteria for acceptable sink conditions), (iii) amount and method of formulation application, (iv) sampling time and test duration, (v) analytical method used for drug quantification in receptor solution. Furthermore, it is required to demonstrate the appropriate discriminatory power of IVPT using the batches with significant alterations compared to the finished product (e.g., by changing the product strength, quantitative composition, CQA and process parameters) [14]. Here, it should be noted that a similar procedure for IVPT is also described in the FDA product-specific guideline (*Draft Guidance on Acyclovir*), but again with more attention to detail regarding the method development, validation, and statistical data analysis [44].

One of the main limitations of the proposed IVPT method is the relatively high number of skin donors required to achieve optimal statistical power for the bioequivalence demonstration. Due to the inherent variability in skin permeability, according to the EMA draft guideline, 12 donors with at least two skin sections per donor are required [14]. The FDA does not define the exact number of donors (i.e., only requires multiple skin donors), but a minimum of 4 replicate skin sections per donor per treatment group is recommended [44]. However, it should be emphasized that, depending on the variability of the obtained data, the number of skin donors should be further increased. In this context, recently, Shin et al. [50] evaluated whether an IVPT method could be used to compare the bioavailability of acyclovir from different commercially available creams. Due to the large inter- and intra-donor variability of IVPT data, authors utilized a novel statistical approach adapting one previously developed to evaluate scaled average bioequivalence (SABE) for highly variable drugs. The implementation of SABE analysis enabled them to capitalize upon the ability of IVPT methodology to sensitively discriminate differences in acyclovir permeation through the skin from any single individual, while compensating for the variability from one individual compared to another [50]. In other words, this statistical approach was shown to improve the power of comparative IVPT studies, thus reducing the number of skin donors (16 donors with four replicates per donor per treatment group) compared to traditional average bioequivalence analysis requiring almost 40 donors. As authors concluded, the IVPT method, followed by an appropriate statistical analysis of the obtained results, is a sensitive and discriminative test that can support the demonstration of bioequivalence for topical semisolid drug products [50].

However, considering that the human skin is usually obtained from plastic surgeries, it could be extremely difficult to procure a sufficient amount of ex vivo skin sections [5]. Therefore, in order to overcome the limitations in supplying excised human skin, animal skin models (most frequently porcine ear skin) have been intensively used in the literature. Although several guidelines recommend the use of animal skin to predict local bioavailability (e.g., SCCS/1358/10 for in vitro assessment of dermal absorption of cosmetic ingredients [52], OECD for bioavailability evaluation of dermal products in 2010 [53]) or systemic absorption (EMA Guideline on quality of transdermal patches in 2014 [54]), due to high variability in skin permeability of different animal models, it is quite difficult to perform a valid comparison between the results obtained across various species [11,51,55]. Therefore, during the last two decades, the scientific community has shown increasing interest in artificial skin surrogates for conducting in vitro permeation studies. Three different types of skin surrogates have been intensively tested, including artificially cultured human skin models (reconstructed human epidermis (e.g., EpiSkin™, EpiDerm™, SkinEthic™, EpiCS^®^, Labcyte model) and the full human skin models (e.g., StrataTest^®^ model, GraftSkin^®^, Vitrolife-Skin™ model), parallel artificial membrane permeability assays (PAMPAs), and artificial membranes based on simple polymeric or lipid models (e.g., Strat-M™) [55]. Although these artificial skin surrogates offer numerous advantages (e.g., defined thickness, composition, ease in handling and storage, and reproducibility in the permeation data), the correlation with the human data is often poor, due to inability to completely recreate the heterogeneous nature of the skin, including cell metabolism and skin appendages. Consequently, skin surrogates are currently recommended for the early screening of different formulations, while human skin should be used for the in vitro permeation testing of finished drug products [55,56].

### 3.2. Stratum Corneum (SC) Sampling

SC sampling (popularly called tape stripping) represents a simple, minimally invasive (skin barrier properties are completely repaired within a few days) technique that involves the sequential removal of superficial skin layers using adhesive tapes [5,9,14]. In case of semisolid products that act on or in the SC, the measurement of drug concentrations in the SC during uptake and elimination phases is directly relevant to characterizing the rate and extent of drug at the site of action. Substantial evidence indicates that the rate and extent of drug disposition in the SC also correlate with those attained into the viable underlying tissues [5,14,57]. Therefore, for semisolid drug products acting beyond the SC, tape stripping may provide a suitable surrogate to characterize the rate and extent of drug absorption to deeper skin layers [5,14,57]. The SC sampling approach was first described in a 1998 FDA Draft Guidance for Industry as a universal method for demonstrating bioequivalence of all topical drug products [58]. This document was withdrawn four years later, due to inconsistency in results found by two independent expert laboratories with commercially available tretinoin gel products [5,9,57]. After 2002, considerable research efforts have been focused on the refinement and improvement of the proposed methodology. The tape stripping procedure recently described in the EMA draft guideline mainly relies on the approach proposed by Professors Richard Guy and Annete Bunge in 2007–2009 (e.g., [59,60]). Thus, in the following section, the most important features of this refined methodology are presented in brief.

Instead of eight time points (four for uptake and four for elimination phase) required to establish the kinetic profile of a drug within the SC according to original FDA draft guidance [58], EMA proposed a simplified, two-time point method, whereby tape stripping should be performed once in the uptake phase and once in the clearance phase [14]. During the pilot study, the optimal uptake time should be established by testing multiple uptake times to detect the time point from which the mass of drug recovered from the SC remains constant (i.e., when diffusional steady state is achieved). The optimal clearance time should be defined by detecting the time point at which at least 25% decrease in the mass of drug recovered from the SC occurs, compared to the one achieved in the uptake phase (should not exceed 48 h to avoid skin desquamation effects) [14]. However, it should be emphasized that the selection of optimal time point has been the subject of various criticism in the literature. For example, according to Rath and coworkers [61], the SC sampling at the time point when the amount of drug in the SC has reached the steady state can mask differences in formulations. For this reason, the approach described in the FDA’s guidance for vasoconstrictor assay was proposed to ensure that the chosen dose duration lies on the sensitive part of the dose-response curve [61]. Further, since the contradictory results obtained between two laboratories following the FDA draft guidance were inter alia attributed to differences in the control of lateral spreading from the application site [60], to minimize inter-site variability, EMA recommends a double template design for the sample application and SC sampling (one template delineating the application area, another delineating the sampling area). Cleaning the skin surface has been recognized as particularly important and has to be carefully validated, by demonstrating the satisfactory recovery (>90%) of the drug formulation removed from the skin surface and the negligible drug content (<10%) recovered by stripping the cleaned skin immediately after application [14]. Unlike withdrawn FDA guidance that required the sequential application and removal of 12 pieces of adhesive tapes [58], EMA defines that the minimum and maximum number of tapes should be established based on transepidermal water loss (TEWL) measurements (tape removal should be stopped when TEWL value exceeded eight times the baseline pre-stripping value) [14]. Likewise, since the lack of assurance of “tape equivalence” between different manufacturers but also within different batches/production years of the same manufacturer, has been identified as one of the major weaknesses for ensuring the appropriate reproducibility of the tape stripping protocol [9], EMA outlines specific requirements that should be met for adhesive tapes. Instead of discarding the first two tapes, as suggested by the withdrawn FDA draft guidance [58], all stripped tapes from each treatment site should be analyzed, whereby the first two tapes should be examined separately, and their contribution to the total amount of the drug recovered should be evaluated [14]. Finally, the number of subjects involved in the study should be justified based on the variability estimated from the pilot study and demonstrated to be statistically relevant. A minimum of 12 subjects should be used for demonstration of equivalence [14]. Since it was previously shown that duplicate application of each formulation reduces the magnitude of variability in tape stripping data and improves its reproducibility [59,62], according to EMA draft guideline, at least two application sites per product (test, comparator and negative controls) per forearm (one for uptake and one for elimination phase) should be involved [14]. Investigating the potential of tape stripping in humans to assess bioequivalence of topical acyclovir cream products (the site of action of acyclovir is beyond the SC, in the basal epidermis), Pensado et al. [57] observed high within-subject standard deviation in the obtained mass per unit area of drug in the SC from the selected reference product. Using SABE methodology proposed for assessing highly variable IVPT data, it was estimated that 10–15 subjects are needed to achieve the statistical power of at least 80%, while traditional average bioequivalence analysis is estimated to require between 15 and 50 subjects [57]. Although widening of the bioequivalence limits has been frequently proposed to reduce the number of subjects and to improve comparison efficiency, it lowers the standard for comparability of the test and reference products. In contrast, SABE analysis with the traditional bioequivalence limit increases the power of the study to an even greater degree than widening of the bioequivalence limits and, therefore, could be more useful for demonstration of bioequivalence of highly variable drugs [57].

Since the protocol proposed in EMA draft guideline is quite cumbersome, Ozdin et al. [62] suggested novel dermatopharmocokinetic approach based on only one dose duration during the uptake phase to generate drug content in SC versus time profiles, whereby each time point corresponds to one stripped layer. Population pharmacokinetics modeling, applying ADAPT^®^ 5 software (Biomedical Simulations Resource, Los Angeles, CA., USA) with maximum likelihood expectation maximization (MLEM) algorithm, was used to fit the obtained data and to estimate the rate and extent of drug absorption or input into the skin. The rational for described concept comprising only one dose duration lies in the fact that bioequivalence assessment is actually the test of formulation performance. The performance of topical semisolid products comprises drug release from formulations and its partitioning into the skin (following partitioning into the SC, drug penetration into deeper skin layers depends on drug properties rather than on formulation performance). The proposed approach based on population pharmacokinetic modeling was deemed successful for topical semisolids, using the approved generic and reference acyclovir creams that were shown to be bioequivalent in an appropriate clinical endpoint study. Although the estimates of the rate and extent of drug absorption with population pharmacokinetic modeling were associated with less inter-individual variability, despite the highly variable tape stripping data, further studies are required to investigate feasibility and the discriminatory power of this approach [62]. Finally, it is interesting to note that recently FDA approved a generic diclofenac sodium topical gel (1%), based on the collective evidence including (i) Q1 and Q2 sameness and physical and structural similarity to the reference product, (ii) an in vivo bioequivalence study with pharmacokinetic endpoints, and (iii) a virtual bioequivalence assessment leveraging dermal physiologically-based pharmacokinetic (PBPK) modeling and simulation instead of a comparative clinical endpoint study in patients. The multi-phase multi-layer (MPML) MechDermA model implemented within the Simcyp Simulator (Certara, Princeton, NJ., USA) was used for PBPK modeling [63,64]. To the best of our knowledge, this is the first ANDA approval utilizing the PBPK modeling to support the bioequivalence of topical semisolid drug products.

## 4. Conclusions

The draft guideline on the quality and equivalence of topical products recently issued by EMA represents a long-awaited regulatory advance regarding alternative approaches for equivalence testing of topical products in lieu of therapeutic equivalence clinical trials. Generally, implementation of an extended pharmaceutical equivalence concept, supported by appropriate in vitro and in vivo methodologies (depending on the complexity of product being studied) will enable a reliable and scientifically driven assessment of bioequivalence of topical generic products. The main identified constraints of the EMA draft guideline which limit its successful translation into practice are mainly related to the quite restrictive acceptance criteria regarding the extended pharmaceutical equivalence, product performance, and efficacy documentation. In this context, it is expected that widening of the acceptance range and/or adoption of proposed statistical approaches, taking into account the intrinsic variability of product being studied will allow more efficient assertion of the product equivalence, simultaneously maintaining the rigorous quality standards. All proposed characterization methods, both in vitro and in vivo, have certain limitations, but they do not have the same limitations, and information from one can complement another. In other words, the collective weight of evidence obtained from comparing product quality and performance is expected to facilitate the development, registration, and ultimately approval of generic semisolid drug products. In this context, it is important to emphasize that due to numerous challenges associated with the experimental analysis of CQA and product transformation after administration, it would be helpful that EMA provides the closer recommendations for methods that should be used for their characterization. Finally, it is reasonable to anticipate that, in the near future, a refined guideline will allow for a significant increase in the availability of multisource generics on the market, which, in turn, will improve patient access to more affordable topical dermatological drug products.

## Figures and Tables

**Table 1:** FDA non-binding product-specific draft guidelines for topical generic semisolid drug products that contain in vitro option for establishing bioequivalence [13].

Drug	Semisolid Dosage Form	Qualitative and Quantitative Sameness Evaluation	Physicochemical Characterization	In Vitro Release Testing	In Vitro Skin Permeation Testing	Additional In Vivo Study	Year
Acyclovir	Ointment	+	+	+			2019
Acyclovir	Cream	+	+	+	+		2016
Bexarotene	Gel	+	+	+	+		2019
Ciprofloxacin hydrochloride	Ointment	+	+	+			2018
Clindamycin phosphate	Gel	+	+	+			2020
Clindamycin phosphate and Tretinoin	Gel	+	+	+			2020
Crisaborole	Ointment	+	+	+	+	PK	2019
Crotamiton	Cream		+				2016
Dapsone	Gel	+	+	+	+	PK	2019
Docosanol	Cream	+	+	+			2017
Doxepin hydrochloride	Cream	+	+	+	+	PK	2019
Gentamicin sulfate	Cream Ointment		+				2017
Hydrocortisone	Cream		+				2017
Ivermectin	Cream	+	+	+	+	PK	2019
Lidocaine	Ointment	+	+				2016
Luliconazole	Cream	+	+	+	+		2018
Metronidazole	Gel	+	+	+			2019
Metronidazole	Cream	+	+	+	+		2019
Nystatin and Triamcinolone acetonide	Cream Ointment		+				2017
Oxymetazoline hydrochloride	Cream	+	+	+	+		2019
Ozenoxacin	Cream	+	+	+	+		2019
Penciclovir	Cream	+	+	+	+		2018
Pimecrolimus	Cream	+	+	+	+		2019
Silver sulfadiazine	Cream	+	+	+			2017
Tacrolimus	Ointment	+	+	+	+		2018
Tretinoin	Gel	+	+	+			2020
Tretinoin	Cream	+	+	+		CES	2020

+ indicates methods recommended by the guidelines; PK—in vivo pharmacokinetic study in humans; CES—clinical endpoint studies.

**Table 2:** IVRT method validation and acceptance criteria according to FDA *Draft Guidance on Acyclovir* [44], EMA *Draft guideline on quality and equivalence of topical products* [14] and recent literature reports [35,36,45,46,47].

Parameter	Short Description	Acceptance Criteria
Membrane inertness	Evaluation of drug binding to membrane should be performed by immersing membrane in solution of drug at concentration relevant to average drug concentration in the receptor solution at the end of the test.	The recovery of drug in solution should be within the range 100% ± 5% [44]
Drug solubility in the receptor medium	Evaluation of drug solubility in the receptor mediums should be performed to confirm its suitability to maintain sink conditions during the study.	Drug concentration in the receptor medium should not exceed 30% of its maximum solubility in the receptor medium [14]
Linearity, precision and reproducibility	The R^2^ value of the in vitro release rate (IVRR) (slope) should be calculated across the sampling times throughout the IVRT study duration, for three IVRT runs with a set of six [44] or 12 [12] diffusion cells on 3 different days. Precision and reproducibility should be assessed from intra-/inter-run data analysis. Intra−/inter-operator precision and reproducibility should be also assessed.	Linearity: Minimum R^2^ > 0.9 across the study duration is required [14,44]. Precision and reproducibility: CV for the intra- and inter-run variability should be <10% [14] or <15% [44]
Sensitivity, specificity and selectivity	Sensitivity should be assessed by comparing the IVRR from the formulations with high (200%), low (50%) and nominal drug concentration (100% of label claim). The specificity should be assessed by determining whether the changes of IVRR are proportional to the different drug concentration in the formulations. The selectivity should be assessed by determining the capability of IVRT method to statistically differentiate the IVRRs from the altered formulations (caused by changes in drug content, CQAs (e.g., drug particle size or product rheological profile), critical manufacturing variables or quantitative excipient composition).	Sensitivity: mean IVRR (low drug concentration) < mean IVRR (nominal drug concentration) < mean IVRR (high drug concentration); Specificity: minimum R^2^ value ≥ 0.90 of the correlation of formulation concentration to average IVRR; Selectivity: CI between altered product formulations should fall outside the limits 90–111% [14] or 75.00–133.33% [44]
Robustness	Robustness testing should include minor variations in the method parameters (mixing rate, temperature, amount of formulation applied and receptor medium composition)	The mean IVRR of runs under altered conditions should be within ±15% of the mean IVRR in the regular parameter setting [44]
Recovery	The recovery should be calculated by dividing the average cumulative amount released at the last point in time with the applied dose in donor chamber.	The dose depletion ≤30% has no influence on the steady-state conditions for drug release [35,36,45,46,47]

## Data Availability

Not applicable.

## References

[1] Kwa M.C., Tegtmeyer K., Welty L.J., Raney S.G., Luke M.C., Xu S., Kong B. (2020). The relationship between the number of available therapeutic options and government payer (medicare part D) spending on topical drug products. Arch. Derm. Res..

[2] Lenn J., Brown M. Cost-Effective Approaches for Successful Generic Dermal Drug Product Authorization. http://staging.ondrugdelivery.com/wp-content/uploads/2018/03/ONdrugDel-SKIN-DRUG-DELI-84-Mar-2018-Medpharm.pdf.

[3] Wu K., Yeoh T., Hsieh Y.L., Osborne D.W., Langley N., Michniak-Kohn B., Osborne D.W. (2019). Quality Assessment of API in Semisolid Topical Drug Products. The Role of Microstructure in Topical Drug Product Development.

[4] Shanley A. Topical Formulation: Moving from Art to Science. APIs, Excipients, and Manufacturing 2016, Supplement to Pharmaceutical Technology 40 (9). https://www.pharmtech.com/view/topical-formulation-moving-art-science.

[5] Raney S.G., Franz T.J., Lehman P.A., Lionberger R., Chen M.L. (2015). Pharmacokinetics-based approaches for bioequivalence evaluation of topical dermatological drug products. Clin. Pharm..

[6] Ilić T., Pantelić I., Lunter D., Đorđević S., Marković B., Ranković D., Daniels R., Savić S. (2017). Critical quality attributes, in vitro release and correlated in vitro skin permeation-In vivo tape stripping collective data for demonstrating therapeutic (non)equivalence of topical semisolids: A case study of “ready-to-use” vehicles. Int. J. Pharm..

[7] Krishnaiah Y.S., Xu X., Rahman Z., Yang Y., Katragadda U., Lionberger R., Peters J.R., Uhl K., Khan M.A. (2014). Development of performance matrix for generic product equivalence of acyclovir topical creams. Int. J. Pharm..

[8] Mohan V., Wairkar S. (2021). Current regulatory scenario and alternative surrogates methods to establish bioequivalence of topical generic products. J. Drug Deliv. Sci. Technol..

[9] Yacobi A., Shah V.P., Bashaw E.D., Benfeldt E., Davit B., Ganes D., Ghosh T., Kanfer I., Kasting G.B., Katz L. (2014). Current challenges in bioequivalence, quality, and novel assessment technologies for topical products. Pharm. Res..

[10] Shah V.P., Yacobi A., Rădulescu F.Ş., Miron D.S., Lane M.E. (2015). A science based approach to topical drug classification system (TCS). Int. J. Pharm..

[11] Miranda M., Sousa J.J., Veiga F., Cardoso C., Vitorino C. (2018). Bioequivalence of topical generic products. Part 2. Paving the way to a tailored regulatory system. Eur. J. Pharm. Sci..

[12] Minghetti P., Musazzi U.M., Casiraghi A., Rocco P. (2020). Old active ingredients in new medicinal products: Is the regulatory path coherent with patients’ expectations?. Drug Discov. Today.

[13] US FDA Product-Specific Guidances for Generic Drug Development. https://www.accessdata.fda.gov/scripts/cder/psg/index.cfm.

[14] (2018). Committee for Medicinal Products for Human Use (CHMP), EMA. Draft Guideline on Quality and Equivalence of Topical Products, CHMP/QWP/708282/2018.

[15] Miranda M., Cardoso C., Vitorino C. (2020). Quality and equivalence of topical products: A critical appraisal. Eur. J. Pharm. Sci..

[16] Simões A., Veiga F., Vitorino C., Figueiras A. (2018). A tutorial for developing a topical cream formulation based on the quality by design approach. J. Pharm. Sci..

[17] Shah V.P., Rădulescu F.Ş., Miron D., Yacobi A. (2016). Commonality between BCS and TCS. Int. J. Pharm..

[18] Chang R.K., Raw A., Lionberger R., Yu L. (2013). Generic development of topical dermatologic products: Formulation development, process development, and testing of topical dermatologic products. AAPS J..

[19] Raghavan L., Brown M., Michniak-Kohn B., Ng S., Sammeta S., Langley N., Michniak-Kohn B., Osborne D.W. (2019). In vitro release tests as a critical quality attribute in topical product development. The Role of Microstructure in Topical Drug Product Development.

[20] Baynes R., Riviere J., Franz T., Monteiro-Riviere N., Lehman P., Peyrou M., Toutain P.L. (2012). Challenges obtaining a biowaiver for topical veterinary dosage forms. J. Vet. Pharm. Ther..

[21] Kumar Sharma P., Panda A., Parajuli S., Badani Prado R.M., Kundu S., Repka M.A., Ureña-Benavides E., Narasimha Murthy S. (2021). Effect of surfactant on quality and performance attributes of topical semisolids. Int. J. Pharm..

[22] Rawat A., Gupta S.S., Kalluri H., Lowenborg M., Bhatia K., Warner K., Langley N., Michniak-Kohn B., Osborne D.W. (2019). Rheological characterization in the development of topical drug products. The Role of Microstructure in Topical Drug Product Development.

[23] Pleguezuelos-Villa M., Merino-Sanjuán M., Hernández M.J., Nácher A., Peris D., Hidalgo I., Soler L., Sallan M., Merino V. (2019). Relationship between rheological properties, in vitro release and in vivo equivalency of topical formulations of diclofenac. Int. J. Pharm..

[24] Kryscio D.R., Sathe P.M., Lionberger R., Yu L., Bell M.A., Jay M., Hilt J.Z. (2008). Spreadability measurements to assess structural equivalence (Q3) of topical formulations-a technical note. AAPS Pharm. Sci. Tech..

[25] Simões A., Miranda M., Cardoso C., Veiga F., Vitorino C. (2020). Rheology by design: A regulatory tutorial for analytical method validation. Pharmaceutics.

[26] Mangas-Sanjuán V., Pleguezuelos-Villa M., Merino-Sanjuán M., Hernández M.J., Nácher A., García-Arieta A., Peris D., Hidalgo I., Soler L., Sallan M. (2019). Assessment of the inter-batch variability of Microstructure Parameters in Topical Semisolids and Impact on the Demonstration of Equivalence. Pharmaceutics.

[27] Miranda M., Cova T., Augusto C., Pais A.A.C.C., Cardoso C., Vitorino C. (2020). Diving into batch-to-batch variability of topical products—A regulatory bottleneck. Pharm. Res..

[28] Xu Z., Mangas-Sanjuán V., Merino-Sanjuán M., Merino V., García-Arieta A. (2020). Influence of inter- and intra-batch variability on the sample size required for demonstration of equivalent microstructure of semisolid dosage forms. Pharmaceutics.

[29] Ocaña J., Monleón-Getino T., Merino V., Peris D., Soler L. (2020). Statistical methods for quality equivalence of topical products. 0.5 Mg/g Betamethasone Ointment as a Case-Study. Pharmaceutics.

[30] Namjoshi S., Dabbaghi M., Roberts M.S., Grice J.E., Mohammed Y. (2020). Quality by design: Development of the quality target product profile (QTPP) for semisolid topical products. Pharmaceutics.

[31] Simões A., Veiga F., Vitorino C. (2020). Progressing towards the sustainable development of cream formulations. Pharmaceutics.

[32] Osborne D.W., Dahl K., Parikh H., Langley N., Michniak-Kohn B., Osborne D.W. (2019). Determination of particle size and microstructure in topical pharmaceuticals. The Role of Microstructure in Topical Drug Product Development.

[33] Kamal N.S., Krishnaiah Y.S.R., Xu X., Zidan A.S., Raney S., Cruz C.N., Ashraf M. (2020). Identification of critical formulation parameters affecting the in vitro release, permeation, and rheological properties of the acyclovir topical cream. Int. J. Pharm..

[34] Murthy N.S. (2016). Critical Quality Attributes (Q3 Characterization) of Topical Semisolid Products. http://www.pharmtech.com/pharmtech-webcasts.

[35] Tiffner K.I., Kanfer I., Augustin T., Raml R., Raney S.G., Sinner F. (2018). A comprehensive approach to qualify and validate the essential parameters of an in vitro release test (IVRT) method for acyclovir cream, 5. Int. J. Pharm..

[36] Miranda M., Pais A.A.C.C., Cardoso C., Vitorino C. (2019). aQbD as a platform for IVRT method development—A regulatory oriented approach. Int. J. Pharm..

[37] Miranda M., Cardoso C., Vitorino V. (2020). Fast Screening Methods for the Analysis of Topical Drug Products. Processes.

[38] SUPAC-SS (1997). Guidance for Industry Nonsterile Semisolid Dosage Forms. Scale-Up and Postapproval Changes: Chemistry, Manufacturing, and Controls; In Vitro Release Testing and In Vivo Bioequivalence Documentation.

[39] Mekjaruskul C., Beringhs A.O., Luo W.C., Xu Q., Halquist M., Qin B., Wang Y., Lu X. (2021). Impact of membranes on in vitro release assessment: A case study using dexamethasone. AAPS Pharm. Sci. Tech..

[40] Patere S., Newman B., Wang Y., Choi S., Mekjaruskul C., Jay M., Lu X. (2020). Influence of in vitro release methods on assessment of tobramycin ophthalmic ointments. Int. J. Pharm..

[41] Ng S.F., Rouse J., Sanderson D., Eccleston G. (2010). A comparative study of transmembrane diffusion and permeation of ibuprofen across synthetic membranes using franz diffusion cells. Pharmaceutics.

[42] Nallagundla S., Patnala S., Kanfer I. (2014). Comparison of in vitro release rates of acyclovir from cream formulations using vertical diffusion cells. AAPS Pharm. Sci. Tech..

[43] Marto J., Baltazar D., Duarte A., Fernandes A., Gouveia L., Militão M., Salgado A., Simões S., Oliveira E., Ribeiro H.M. (2015). Topical gels of etofenamate: In vitro and in vivo evaluation. Pharm. Dev. Technol..

[44] U.S (2016). FDA Draft Guidance on Acyclovir. https://www.accessdata.fda.gov/drugsatfda_docs/psg/Acyclovir_topical%20cream_RLD%2021478_RV12-16.pdf.

[45] Purazi P., Rath S., Ramanah A., Kanfer I. (2020). Assessment of “Sameness” and/or Differences between Marketed Creams Containing Miconazole Nitrate Using a Discriminatory in vitro Release Testing (IVRT) Method. Sci. Pharm..

[46] Mudyahoto N.A., Rath S., Ramanah A., Kanfer I. (2020). In Vitro release resting (IVRT) of topical hydrocortisone acetate creams: A novel approach using positive and negative controls. Dissolution Technol..

[47] Rath S., Kanfer I. (2020). A validated IVRT method to assess topical creams containing metronidazole using a novel approach. Pharmaceutics.

[48] Habjanič N., Kerec Kos M., Kristan K. (2020). Sensitivity of different in vitro performance tests and their in vivo relevance for calcipotriol/betamethasone ointment. Pharm. Res..

[49] (1995). US FDA Guidance for Industry: Topical Dermatologic Corticosteroids: In Vivo Bioequivalence. https://www.fda.gov/media/70931/download.

[50] Shin S.H., Rantou E., Raney S.G., Ghosh P., Hassan H., Stinchcomb A. (2020). Cutaneous pharmacokinetics of acyclovir cream 5% products: Evaluating bioequivalence with an in vitro permeation test and an adaptation of scaled average bioequivalence. Pharm. Res..

[51] Miranda M., Sousa J.J., Veiga F., Cardoso C., Vitorino C. (2018). Bioequivalence of topical generic products. Part 1: Where are we now?. Eur. J. Pharm. Sci..

[52] SCCS/1358/10 Basic Criteria for in Vitro Assessment of Dermal Absorption of Cosmetic Ingredient. https://op.europa.eu/en/publication-detail/-/publication/91793089-8206-4975-a6c9-078770655851.

[53] OECD (2010). OECD Guidance Notes on Dermal Absorption Draft 22 October 2010.

[54] (2014). Guideline on Quality of Transdermal Patches.

[55] Neupane R., Boddu S.H.S., Renukuntla J., Babu R.J., Tiwari A.K. (2020). Alternatives to biological skin in permeation studies: Current trends and possibilities. Pharmaceutics.

[56] Zsikó S., Csányi E., Kovács A., Budai-Szűcs M., Gácsi A., Berkó S. (2020). Novel in vitro investigational methods for modeling skin permeation: Skin PAMPA, Raman mapping. Pharmaceutics.

[57] Pensado A., Chiu W.S., Cordery S.F., Rantou E., Bunge A.L., Delgado-Charro M.B., Guy R.H. (2019). Stratum corneum sampling to assess bioequivalence between topical acyclovir products. Pharm. Res..

[58] (1998). Guidance for Industry: Topical Dermatological Drug Product NDAs and ANDAs—In Vivo Bioavailability, Bioequivalence, In Vitro Release, and Associated Studies. Draft Guidance.

[59] Navidi W., Hutchinson A., N’Dri-Stempfer B., Bunge A. (2008). Determining bioequivalence of topical dermatological drug products by tape-stripping. J. Pharm. Pharm..

[60] N’Dri-Stempfer B., Navidi W.C., Guy R.H., Bunge A.L. (2008). Optimizing metrics for the assessment of bioequivalence between topical drug products. Pharm. Res..

[61] Rath S., Ramanah A., Bon C., Kanfer I. (2020). Application of a dermatopharmacokinetic (DPK) method for bioequivalence assessment of topical metronidazole creams. J. Pharm. Pharm. Sci..

[62] Ozdin D., Kanfer I., Ducharme M.P. (2020). Novel Approach for the bioequivalence assessment of topical cream formulations: Model-based analysis of tape stripping data correctly concludes BE and BIE. Pharm. Res..

[63] Tsakalozou E., Babiskin A., Zhao L. (2021). Physiologically-based pharmacokinetic modeling to support bioequivalence and approval of generic products: A case for diclofenac sodium topical gel, 1%. CPT Pharm. Syst. Pharm..

[64] Orange Book: Approved Drug Products with Therapeutic Equivalence Evaluations. https://www.accessdata.fda.gov/scripts/cder/ob/index.cfm.

